# Study of the Luminescence Decay of a Semipolar Green Light-Emitting Diode for Visible Light Communications by Time-Resolved Electroluminescence

**DOI:** 10.1021/acsphotonics.2c00414

**Published:** 2022-07-05

**Authors:** Jack Ivan Holly Haggar, Suneal S. Ghataora, Valerio Trinito, Jie Bai, Tao Wang

**Affiliations:** Department of Electronic and Electrical Engineering, The University of Sheffield, Sheffield S1 3JD, U.K.

**Keywords:** InGaN, GaN, LED, TREL, TRPL, VLC

## Abstract

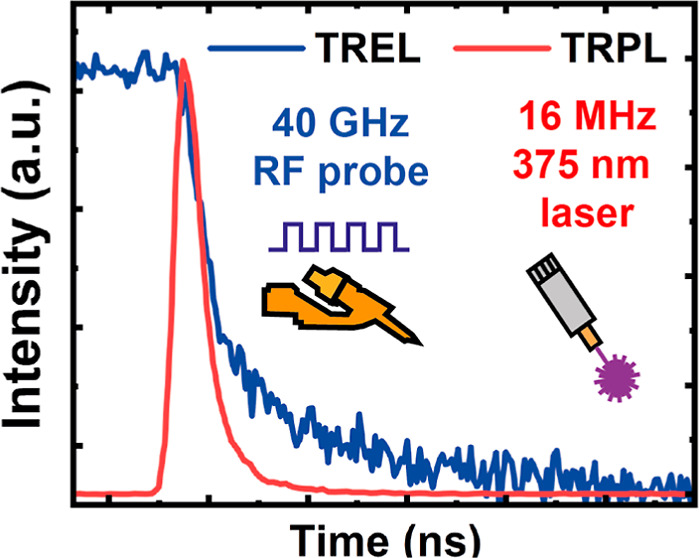

Time-resolved photoluminescence (TRPL) is often used to study the excitonic dynamics of semiconductor optoelectronics such as the carrier recombination lifetime of III-nitride light-emitting diodes (LEDs). However, for any real-world application that requires LEDs under electrical injection, TRPL suffers an intrinsic limitation due to the absence of taking carrier transport effects into account. This becomes a severe issue for III-nitride LEDs used for visible light communication (VLC) since the modulation bandwidth for VLC is determined by the overall carrier lifetime of an LED, not just its carrier recombination lifetime. Time-resolved electroluminescence (TREL), which can characterize the luminescence decay of an LED under electrical injection to simulate real-world conditions when used in practical applications, is required. Both TRPL and TREL have been carried out on a semipolar LED and a standard c-plane LED (i.e., polar LED) both in the green spectral region for a comparison study. The (11-22) green semipolar LED exhibits much faster differential carrier lifetimes than the c-plane LED. In addition to a fast exponential component and a slow exponential component of 0.40 and 1.2 ns, respectively, which are similar to those obtained by TRPL, a third lifetime of 8.3 ns due to transport-related effects has been obtained from TREL, which has been confirmed by capacitance measurements. It has been found that the overall carrier lifetime of a c-plane LED is mainly limited by RC effects due to a junction capacitance, while it is not the case for a semipolar LED due to intrinsically reduced polarization, demonstrating the major advantages of using a semipolar LED for VLC.

## Introduction

There is an increasing demand for developing a visible light communication (VLC) technology that is based on a visible emitter [either a light-emitting diode (LED) or a laser diode] as a transmitter, an emerging wireless communication technology offering a complementary approach to radio frequency (RF)-based Wi-Fi and 5G. As a result of using visible light, whose wavelength is much shorter than those of RF, the frequency bandwidth is more than 3 orders of magnitude larger than those for RF. It has been predicted that VLC provides a long-term solution to the looming RF “spectrum crunch” due to a substantial increase in data demand.^1^

A data transmission rate is largely determined by the bandwidth of a transmitter and the signal-to-noise ratio of a receiver. A high data transmission rate requires a visible emitter with both high output power and a short carrier lifetime. Time-resolved photoluminescence (TRPL) is a powerful tool for characterizing a carrier recombination lifetime but is fundamentally confined to the active region of an emitter and does not contain information about carrier transport through the emitter structure, active region carrier density, or performance when considering real-world LED applications under biasing conditions (i.e., electrical injection).^2−4^ Therefore, it is crucial to explore a method that allows us to study the carrier dynamics of an emitter under biasing conditions for VLC applications.

In general, the modulation bandwidth of an LED (labeled as *f*) is inversely proportional to the overall carrier lifetime of an LED (labeled as τ), that is, *f* ∝ 1/τ, which can be described by eq 1 given below
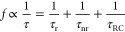
1where τ_r_ is the lifetime due to radiative recombination and τ_nr_ the lifetime due to nonradiative recombination. It is worth highlighting that the third component labeled as τ_RC_ is the lifetime due to the junction capacitance of an emitter, that is, the so-called RC effects (i.e., resistance labeled as R and junction capacitance labeled as C). The junction capacitance of an emitter sensitively depends on its dimension. The dimension of a standard III-nitride LED is >300 μm × 300 μm. Generally speaking, an emitter with an area of <100 μm^2^ is not limited by τ_RC_ in terms of overall carrier lifetime and modulation bandwidth for VLC applications.^5−8^ However, broader-area LEDs are desirable in terms of easier fabrication, higher optical power, and cheaper integration solutions, where it is essential to consider τ_RC_ if they are used for VLC applications.

τ_r_ and τ_nr_ are normally studied and can be separated by means of using temperature-dependent TRPL measurements with the assumption that τ_nr_ can be safely negligible at a low temperature.^4^ This method, while being sufficient for the optical investigation of an emitter under optical pumping conditions, does not tell the whole story for an emitter under biasing conditions since TRPL completely neglects carrier transport effects. Moreover, since TRPL is performed under optical pumping, it is challenging to quantify the carrier density in the active region of an emitter. Therefore, τ_r_ only provides an approximate characterization since τ_r_ = 1/*BN*, where *B* is the recombination coefficient and *N* is the carrier density.^9^ There is an alternative to TRPL, namely, time-resolved photocurrent, which has been applied to the investigation of transport effects of GaInP solar cells.^10^ For InGaN LEDs under electrical injection for VLC applications, electroluminescence (EL) measurements are essential.

In an ideal case, the luminescence decay of an LED should be characterized under electrical injection to simulate real-world conditions when used in practical applications. Therefore, time-resolved electroluminescence (TREL) measurements as a function of bias will allows us to accurately investigate the decay dynamics of an LED for practical applications, which is particularly important for VLC applications. TREL demonstrates two major benefits. The excitation signal (electrically injected square wave with an ultrafast falling edge) used for TREL is described as a small perturbation compared to the DC bias used to electrically drive an LED, that is, d*R*/d*N*. The resulting lifetimes measured are therefore *differential lifetimes* instead of carrier recombination lifetimes measured from TRPL, with the latter being around 2–3 times smaller than the former.^10,11^ The carrier density in the active region can be then calculated by simply integrating the differential lifetime across the thickness of the active region, from which a physical model can be applied to the resulting response.

The differential carrier lifetime has been studied extensively using various methods for optoelectronics with most reports based on vector network analyzers, where both the impedance and the optical modulation response of an LED can be measured and both responses can be fit simultaneously to a physical model.^11−15^ The optical receivers are usually silicon-based photodetectors with either integrated transimpedance amplifiers or separate operational amplifiers that intrinsically suffer from low responsivity in the visible spectral range (e.g., 0.1 A/W at 450 nm). As III-nitride emitters used as transmitters for VLC applications are approaching the limit of these detectors,^16−18^ recent efforts have been focused on finding new alternatives for the receivers in VLC characterization systems. By means of using a combination of time-correlated single photon counting (TCSPC) electronics and hybrid photomultiplier tubes (PMTs), which are similar to what are used in TRPL, we can establish an electrically injected time-resolved system that exhibits high sensitivity with impulse responses of <50 ps compared with conventional photodetectors used in frequency domain systems.

There are also key issues associated with commercially available LEDs used for VLC. Commercially available white LEDs are typically fabricated by using polar orientated III-nitride blue LEDs grown on c-plane substrates coupled with yellow phosphors as conversion layers, where such am LED suffers greatly from strain-induced piezoelectric fields across the InGaN/GaN multiple quantum well (MQW) emitting region.^19,20^ The blue LEDs experience a reduced overlap between the electron–hole wavefunctions, leading to a long radiative recombination time and thus low quantum efficiency, that is, the so-called quantum confined Stark effect (QCSE). The color conversion process is also very slow, resulting in increased response lifetimes.^21^ Replacing the phosphor layers by longer wavelength emitters such as yellow or red LEDs results in an even larger strain across the InGaN MQWs and therefore an enhanced QCSE, which further increases the carrier recombination lifetime. Growing III-nitride LEDs along a semipolar or nonpolar orientation, such as the LEDs grown on (11-22) semipolar substrates, has been proposed to naturally minimize or even eliminate the QCSE, effectively increasing the recombination rate.^22^ The (11-22) orientation also facilitates the enhancement of indium incorporation into InGaN, enabling longer-wavelength emitters along with a reduction in polarization.^23^

Recently, we have demonstrated record breaking modulation bandwidths and multi-gigabyte per second data transmission rates for our large-area semipolar LEDs up to the amber spectral region.^11,25^ In this paper, we explore further into the nature of semipolar LEDs due to reduced polarization and their benefits for VLC. In this paper, both TREL and TRPL measurements have been performed on two different kinds of green LEDs, one grown on c-plane sapphire and the other grown on our high-quality semipolar (11-22) GaN overgrown on m-plane sapphire substrates.^24^ Interestingly, we have found that even though RC measurements suggest that both the c-plane LED and the semi-polar LED should be limited by τ_RC_, only the c-plane LED suggests that τ_RC_ is a limiting factor, while the TREL data from the semipolar LED are comparable to their TRPL results. Our results have shown that the semipolar LED demonstrates an increased recombination rate compared with the c-plane LED. More importantly, it has been found out that RC effects exhibit less contribution to the overall carrier lifetime for the semipolar LED than that for the c-plane LED, demonstrating the major advantage of using semipolar LEDs for VLC applications compared with c-plane LEDs.

## Results and Discussion

**Figure 1:**
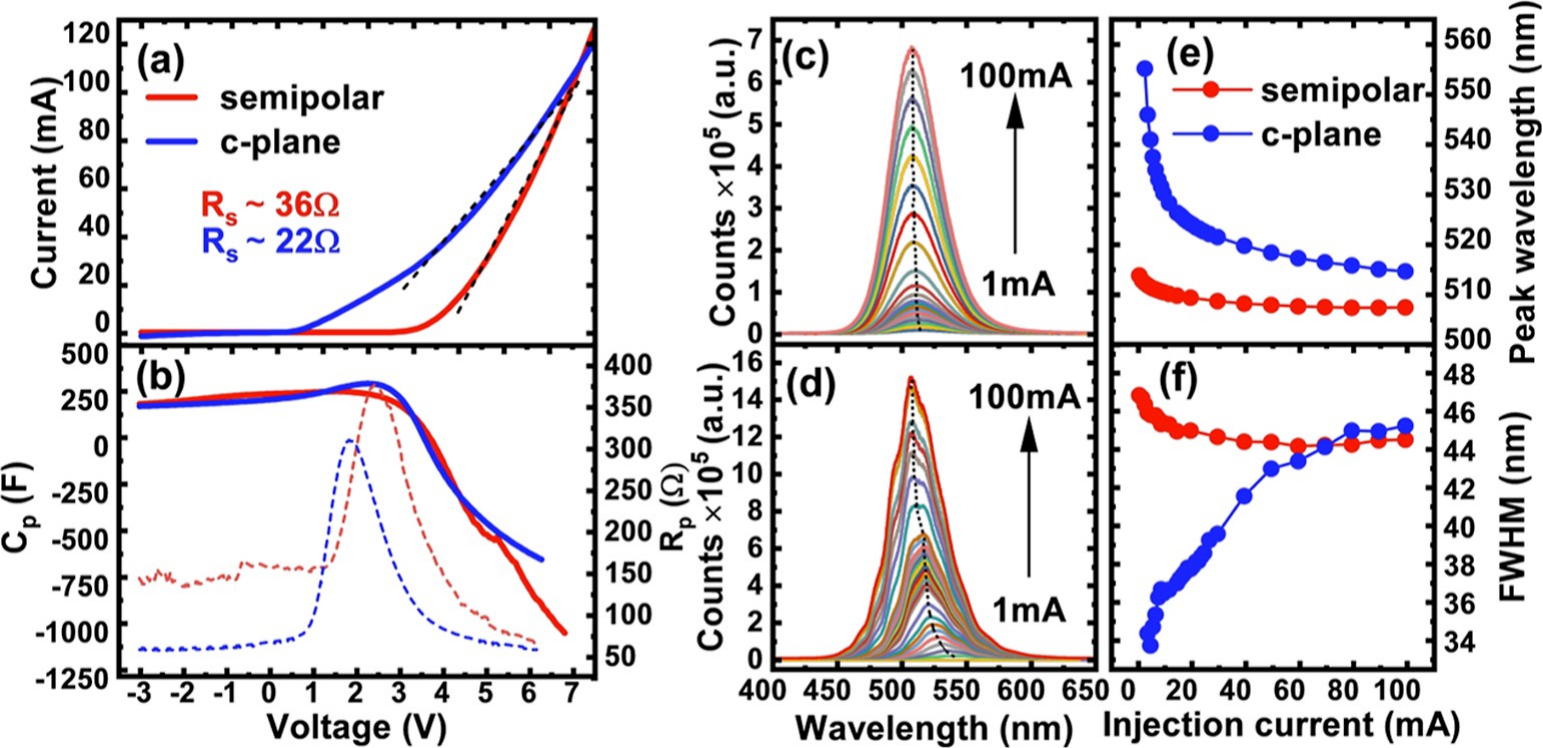
Electrical and optical characteristics of the semipolar LED and the c-plane LED: *I*–*V* curves and series resistance (a); capacitance (solid) and resistance (dashed) measured using an LCR meter in a parallel circuit mode at 1 MHz (b); EL spectra of the semipolar LED as a function of injection current (c); EL spectra of the c-plane LED as a function of injection currents (d); peak wavelength shift of both LEDs as a function of injection current (e); and FWHM of the EL spectra of the both LEDs as a function of injection current (f).

Figure 1a shows the current–voltage (*I*–*V*) characteristics of both the semipolar LED and the c-plane LED, indicating turn-on voltages (measured at 20 mA) of 4.3 and 2.5 V with corresponding series resistances of 36 and 22 Ω for the semipolar LED and the c-plane LEDs, respectively. Figure 1b exhibits the capacitance and resistance of these two LEDs measured using a precision LCR meter at 1 MHz in a parallel circuit mode, where the capacitance of an LED is given by its depletion capacitance below 0 V.^27^ Therefore, the RC time constant of each LED can be obtained using the series resistance and the depletion capacitance under a negative bias. In this case, capacitance values of 194 and 173 pF have been measured at −2 V, corresponding to RC time constants of 7.0 and 3.8 ns for the semipolar LED and the c-plane LEDs, respectively. It is worth highlighting that these values are an approximate for the real RC lifetime and only serve as a lower limit due to the nonlinearity of the devices. Based purely on these measurements, however, if the carrier lifetime is in fact found to be dominated by the RC lifetime (as the typical size of a standard LED is >300 × 300 μm^2^) when electrically injected, we would expect the c-plane LED to exhibit a faster decay time when measured on the time-resolved system.

Figure 1c,d shows the EL spectra of the two LEDs measured as a function of the injection current for the semipolar LED and the c-plane LEDs, respectively. At 100 mA, both LEDs exhibit similar peak wavelengths of 507 and 514 nm for the semipolar LED and the c-plane LEDs, respectively. Due to the reduced QCSE, the emission wavelength shift of the semipolar LED is only 7 nm, compared with 40 nm for the c-plane LED when measured between 1 and 100 mA, as shown in Figure 1e. Similarly, Figure 1f displays that the full width at half maximum (FWHM) of the semipolar LED experiences a 3 nm narrowing at the injection current of up to around 60 mA due to the naturally reduced polarization and then broadens slightly at higher injection currents mainly due to band filling effects. In contrast, the c-plane LED experiences an 11 nm broadening between 1 and 100 mA due to QCSE and band filling effects. This has been often observed in III-nitride c-plane LEDs.

**Figure 2:**
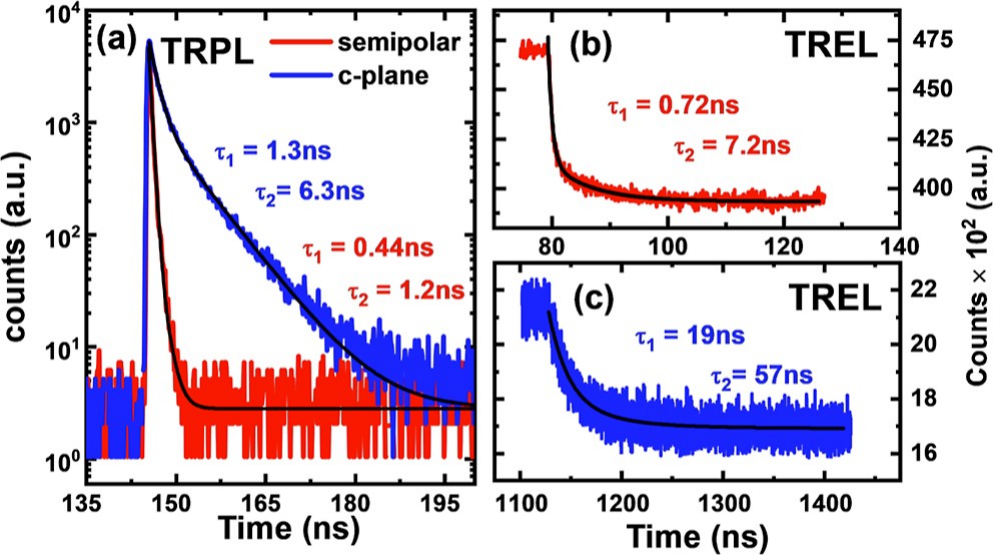
TRPL decay traces and fitting with a bi-exponential model showing extracted lifetimes for both the semipolar LED and the c-plane LED (a) and TREL decay traces for the semipolar LED (b) and c-plane (c) LED with the same bi-exponential model applied with associated extracted lifetimes.

Figure 2a shows the results of TRPL measurements performed on both samples at room temperature. A bi-exponential model is typically employed to fit the decay trace of InGaN, namely, a fast exponential component and a slow exponential component to fit the decay traces.^2−4,28,29^ This behavior is usually due to two radiative recombination channels contributing to the decay. The exciton binding energy of GaN is large (>26 meV), meaning that room-temperature excitons and carrier localization effects are the main factors contributing toward the bi-exponential nature of the decay.^30−32^ The extracted lifetimes from the TRPL data are 0.44 and 1.3 ns for τ_1_ and 1.2 and 6.3 ns for τ_2_ corresponding to the semipolar and c-plane LEDs, respectively. It is clear that the semipolar LED exhibits much faster lifetimes for both fast and slow components, which are attributed to a reduction in QCSE, allowing an enhanced recombination rate. However, for VLC applications that require fast LED switching speeds under electrical injection, TRPL results are not enough to confirm high performance as TRPL neglects carrier transport effects through the LED structure. We therefore extend this measurement further by electrically injecting the device and observing the EL decay dynamics over time. Figure 2b,c show the results of TREL decay traces of the two LEDs both measured at room temperature, which will be discussed later on.

**Figure 3:**
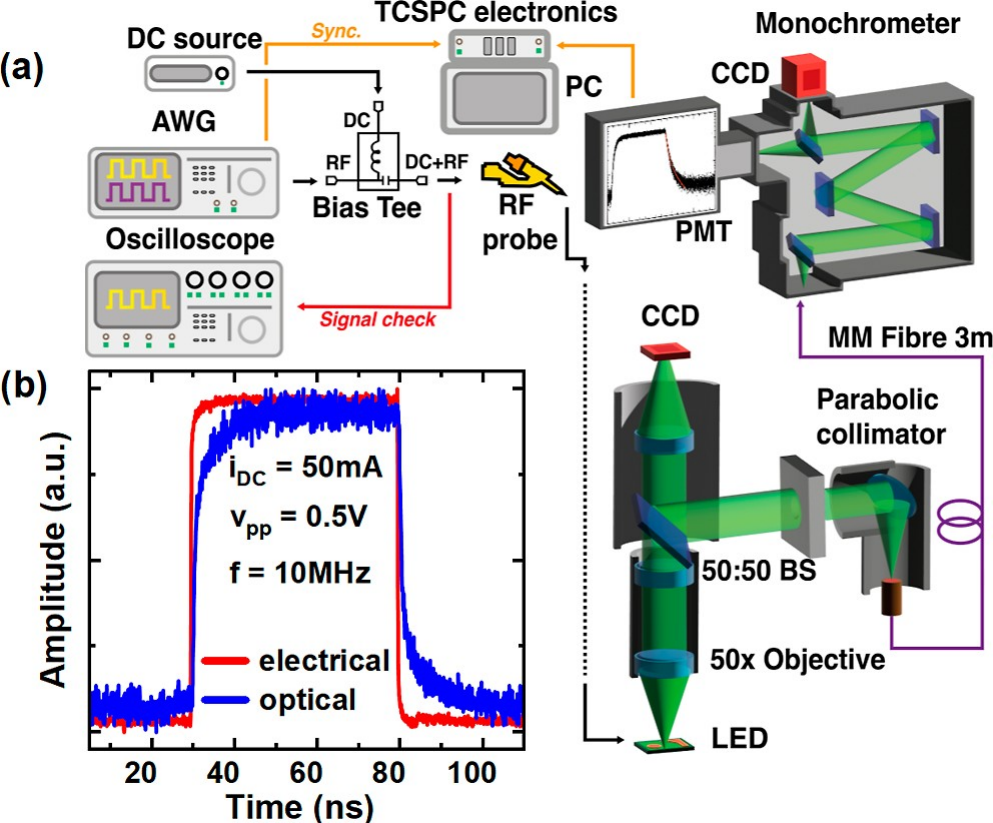
Schematic of our TREL system (a) and typical electrical input signal (red) from an AWG measured on an RF oscilloscope and the measured output (blue) from a PMT (b).

Figure 3a shows the experimental setup for our TREL system. Figure 3b shows a typical example for an electrical input waveform generated by an arbitrary wave generator (AWG) as an excitation source and the resulting histogram waveform acquired over time from a PMT detector displaying the LED recombination dynamics. For the semipolar LED, a 500 mV (peak-to-peak) waveform with a repetition rate of 10 MHz (100 ns) is used as an excitation source, while an identical but less frequent 1 MHz (1000 ns) source is used for the c-plane LED. The lower frequency for the c-plane LED is necessary for the device to reach a steady state before measuring decay dynamics. In any time-resolved system, the instrument response function (IRF) needs to be taken into consideration. Since it is very difficult to directly measure the IRF of a system such as this, which takes both the electrical and optical sides into account simultaneously (this would require a probe-able, electrically injected emitter operating at >500 nm with picosecond response times), we outline the response times of each major component in the system in Table 1, which should serve as a good approximation of the IRF. The IRF is approximated to be ∼67 ps, calculated using the sum of the squares of each component’s response time.

**Table 1:** Response Time of Each Component of Our TREL System to Estimate the IRF

component	response time (ps)
AWG	22
Bias Tee	30
TCSPC electronics	6.5
RF probe	25
PMT	<50
RF cables	38

Figure 2b,c shows the TREL traces of the two LEDs and their corresponding fitting results for a typical waveform biased at 50 mA for both LEDs. Initially, a bi-exponential model similar to that we used for TRPL was employed to fit the decay curves of both LEDs, aiming to approximately compare the two types of measurements. In this case, the extracted lifetimes from the TREL data are 0.72 and 19 ns for τ_1_ and 7.2 and 57 ns for τ_2_ corresponding to the semipolar LED and the c-plane LEDs, respectively. This direct comparison shows that there are much larger differences between the two LEDs than for TRPL, with the semipolar LED experiencing similar but slightly higher lifetimes than on TRPL and the c-plane LED showing much higher lifetimes.

In addition to the bi-exponential behavior commonly observed in InGaN photoluminescence decay stemming from different radiative recombination channels such as excitonic contributions and carrier localization, a slower third term encompassing various carrier transport-related recombination mechanisms in the cladding and barrier layers, defects, and junction capacitance-related effects is added for TREL measurements.

**Figure 4:**
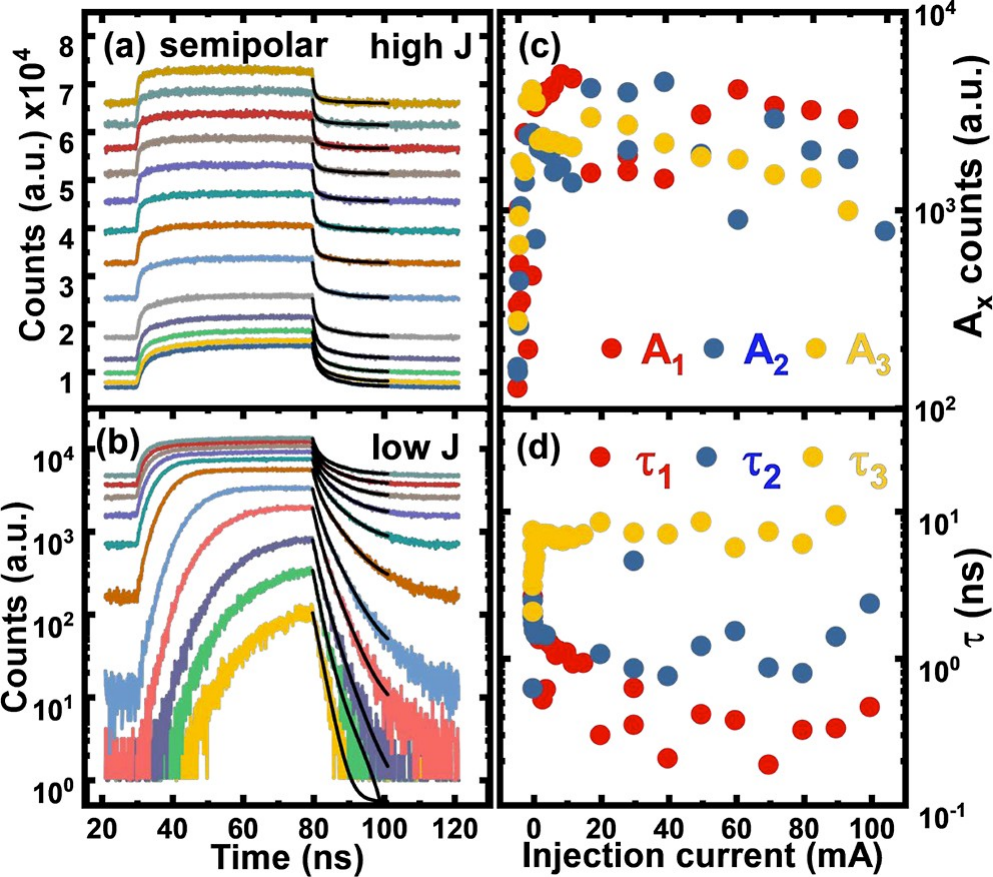
TREL decay traces as a function of injection current for the semipolar LED at high injection (a) and low injection (b). Tri-exponential model fitted to the decay profiles with corresponding amplitudes (c) and extracted time constants (d) as a function of injection current.

Figure 4a,b shows the TREL traces of the semipolar LED measured under high and low injection currents, both at room temperature, respectively, where the tri-exponential fitting given below is used for each data set.
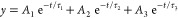
2where τ_1_, τ_2_, and τ_3_ refer to the fastest, fast, and slow components along with their corresponding amplitude coefficients *A*_1_, *A*_2_, and *A*_3_, respectively. Each data set is composed of a different DC bias ranging from 50 μA to 100 mA, and the effect of this DC bias is apparent when observing the baseline counts for each curve. A 500 mV excitation source is added on top of each DC bias using a bias tee so that we can measure recombination dynamics from a steady state as a function of injection current. Between 50 and 200 μA, the LED does not reach a steady state and exhibits extremely fast fall times after the excitation source from the AWG is switched off. This may be due to the excitation source being comparatively much larger than the DC bias level, resulting in the excitation source negatively biasing the device and briefly creating an artificial current shaping circuit that introduces other dominant recombination mechanisms such as carrier sweep out, exceeding the response time of the system. Therefore, under very low injection current, these mechanisms are out of the scope of this study.

Figure 4c,d shows the extracted lifetimes of the semipolar LED obtained using the tri-exponential model. Above 3 mA, there are three clear lifetimes associated with the EL decay of the semipolar LED. In general, τ_1_, τ_2_, and τ_3_ decrease with the increasing injection current as expected since increasing the current density increases the recombination rate and thus the carrier lifetime. τ_3_, the slowest component, seems to be the most dominant lifetime at lower injection current but then slowly decreases in its contribution with the increasing current, as indicated by Figure 4d. At higher injection, τ_1_ becomes the most dominant component, followed closely by τ_2_ and then τ_3_. For example, at 50 mA, the extracted lifetimes are 0.40, 1.2, and 8.3 ns for τ_1_, τ_2_, and τ_3_, respectively. The extracted lifetimes for τ_1_ and τ_2_ are of the same magnitude as those obtained from the TRPL measurements and comparable to other published data on semipolar LEDs.^11,33^ This suggests that this device is not limited by RC effects. The extracted lifetime τ_3_ is responsible for carrier transport effects as it is comparable with the LCR meter measurement, that is, the approximation for the RC lifetime.

**Figure 5:**
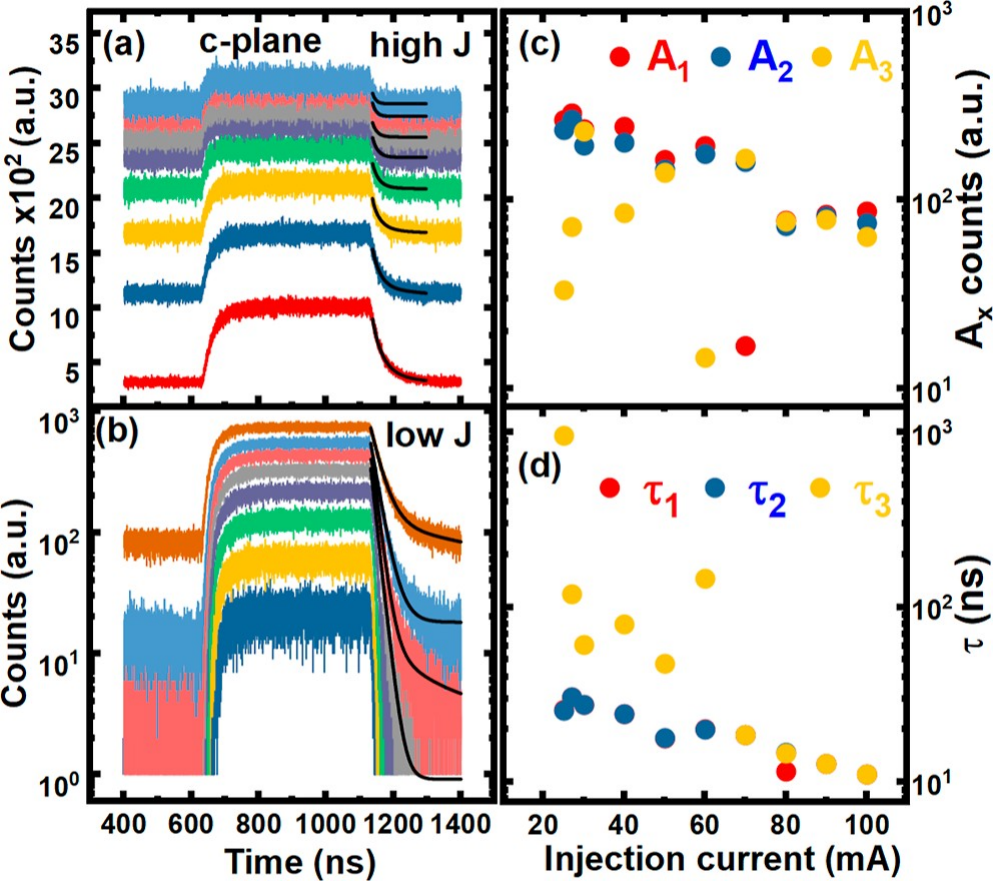
TREL decay traces as a function of injection current for the c-plane LED at high injection (a) and low injection (b). Tri-exponential model fitted to the decay profiles with corresponding amplitudes (c) and extracted time constants (d) as a function of injection current.

Figure 5a,b shows results obtained by applying the same tri-exponential model to the c-plane LED under high and low injection conditions, respectively. Figure 5c displays the amplitude of each component as a function of injection current, while Figure 5d indicates the lifetime of each component as a function of injection current. τ_1_ and τ_2_ converge toward a single lifetime of around 12 ns below 60 mA, while τ_3_ is a much slower component, exhibiting values between 0.1 and 1 μs, as shown in Figure 5d. At higher injection, all three lifetimes converge toward a single lifetime that is, a mono-exponential decay, slowly decreasing to 10 ns at 100 mA. LEDs that typically experience a mono-exponential decay are essentially like an RC circuit since the fall time is limited by transport effects. Interestingly, the RC characteristics measured on this device would suggest that the c-plane LED should perform better than the semipolar LED when measuring dynamics. This clearly is not the case, and the reduction in the QCSE present on the semipolar LED has a much more dominant effect on the recombination lifetime and resulting decay dynamics than the RC measurements, which are expected to be the dominant lifetime with LEDs of this size.

## Conclusions

We have demonstrated the importance of using TREL measurements over TRPL measurements for VLC applications as it is impossible to use TRPL to study transport effects through a LED structure. A detailed comparison study has been conducted by performing TREL and TRPL on a semipolar LED and a c-plane LED both with an emission wavelength in the green spectral region. By performing TREL measurements under bias conditions, the transport effect on carrier lifetime has been studied. The semipolar LED was found to follow closely a tri-exponential decay, with differential carrier lifetimes τ_1_ and τ_2_ contributing toward radiative and nonradiative recombination, respectively, and τ_3_ encapsulating transport effects, while the c-plane LED was reduced to a mono-exponential decay, indicating that τ_RC_ is a limiting factor.

## Methods

### LED Fabrication

Two different kinds of green LEDs have been used in the present study: one standard LED on c-plane sapphire and one (11-22) semipolar LED on our high-quality semipolar (11-22) GaN overgrown on m-plane sapphire.^24,26,34^ The c-plane LED consists of seven periods of In_0.25_Ga_0.75_N/GaN MQW structures (well: 2.5 nm and barrier:13.5 nm). For the semipolar LED, please refer to our previously published papers,^24,26,34^ where a 3 nm single quantum well sandwiched between two 9 nm thick quantum barriers was used as an emitting region for the semipolar LED, and the nominal indium content is 29%. LEDs with a typical dimension of 330 × 330 μm^2^ have been fabricated using a standard photolithography and dry etching method. A 100 nm thick ITO film was deposited on the top of the device to form a transparent p-contact and then annealed using rapid thermal annealing (RTA). A Ti/Al/Ti/Au stack was thermally evaporated onto the *n*-GaN to form the n-contact. Finally, Ti/Au was deposited on both contacts to form the p- and n-electrodes.

### TREL Measurements

An excitation signal consisting of a 10 MHz (1 MHz for the c-plane LED) square wave with an amplitude of 500 mV (peak-to-peak) is created using an AWG (Tektronix AWG70002A). An identical waveform is created on the adjacent AWG channel and used as a trigger for a TCSPC timing electronics system (Becker & Hickl Simple-Tau 130). The excitation signal is combined with a DC bias from a Keithley 2400 power supply through a 12 GHz bias tee (Tektronix PSPL5575A). A 40 GHz RF probe (FormFactor ACP40-GS300RC) is then used to deliver the resulting RF + DC signal to the LED under test. To monitor the input electrical pulse that includes the effect from the cables, bias tee, and so on, the signal is first connected to a 6 GHz oscilloscope (Tektronix DPO70604C) before the LED. After initial checks, the resulting modulated LED emission is collected through a 50 × magnification, 0.42 NA infinity corrected objective. A 50:50 beam splitter is used to split half the collimated light onto a charge-coupled device (CCD) camera (for aligning, probing, etc.) using a lens tube, while the other half is coupled into a multimode fiber using a parabolic collimator. The light is then dispersed through diffraction grating in a monochromator and then focussed onto a hybrid PMT for TCSPC measurements. A flip mirror is used to switch between the PMT and a CCD for spectral measurements. Timing synchronization is achieved by combining TCSPC electronics with the falling edge of the trigger signal from the AWG and hybrid PMT measurements.

### TRPL Measurements

A pulsed 375 nm laser diode with a 50 ps pulse and a repetition rate of 16 MHz is used as an excitation source. The laser spot size is focused to a 2 μm diameter using an NUV 0.43NA infinity corrected objective lens. The emission is collected through the same lens and coupled into a multimode fiber. The light is then dispersed through a monochromator before focusing onto a hybrid PMT. The overall system has an approximate timing resolution of 15 ps and an instrument response time of 150 ps, which is deconvolved from each measurement..
